# Juvenile Selenium Deficiency Impairs Cognition, Sensorimotor Gating, and Energy Homeostasis in Mice

**DOI:** 10.3389/fnut.2021.667587

**Published:** 2021-05-07

**Authors:** Victor W. Kilonzo, Alexandru R. Sasuclark, Daniel J. Torres, Celine Coyle, Jennifer M. Pilat, Christopher S. Williams, Matthew W. Pitts

**Affiliations:** ^1^Department of Cell and Molecular Biology, University of Hawaii, Honolulu, HI, United States; ^2^Pacific Biosciences Research Center, University of Hawaii at Manoa, School of Ocean and Earth Science and Technology (SOEST), Honolulu, HI, United States; ^3^Department of Medicine and Cancer Biology, Vanderbilt University School of Medicine, Nashville, TN, United States

**Keywords:** selenium, sensorimotor gating, cognition, energy metabolism, neurodevelopment

## Abstract

Selenium (Se) is an essential micronutrient of critical importance to mammalian life. Its biological effects are primarily mediated via co-translational incorporation into selenoproteins, as the unique amino acid, selenocysteine. These proteins play fundamental roles in redox signaling and includes the glutathione peroxidases and thioredoxin reductases. Environmental distribution of Se varies considerably worldwide, with concomitant effects on Se status in humans and animals. Dietary Se intake within a narrow range optimizes the activity of Se-dependent antioxidant enzymes, whereas both Se-deficiency and Se-excess can adversely impact health. Se-deficiency affects a significant proportion of the world's population, with hypothyroidism, cardiomyopathy, reduced immunity, and impaired cognition being common symptoms. Although relatively less prevalent, Se-excess can also have detrimental consequences and has been implicated in promoting both metabolic and neurodegenerative disease in humans. Herein, we sought to comprehensively assess the developmental effects of both Se-deficiency and Se-excess on a battery of neurobehavioral and metabolic tests in mice. Se-deficiency elicited deficits in cognition, altered sensorimotor gating, and increased adiposity, while Se-excess was surprisingly beneficial.

## Introduction

Selenium (Se) is an essential trace element in mammals, of which both deficiency and excess can have detrimental effects on health (1). Se supplementation within a narrow range optimizes activity of Se-dependent antioxidant enzymes that incorporate Se co-translationally in the form of selenocysteine. Se also counteracts the toxicity of certain heavy metals, such as arsenic, lead, and mercury (2, 3). Deficient Se intake impairs thyroid hormone metabolism and reduces activity of the antioxidant enzymes, glutathione peroxidase and thioredoxin reductase (4). In contrast, Se can be detrimental at high doses, with documented neurotoxic effects (5).

Se-deficiency is estimated to occur in roughly 10% of the world's population and is observed predominantly in regions with low soil Se-content, such as Scandinavia, New Zealand, and Northeast China (6). Furthermore, future climate change is predicted to decrease soil Se content in agricultural regions and augment the prevalence of Se-deficiency worldwide (7). Common symptoms associated with Se-deficiency include hypothyroidism, cardiomyopathy, compromised immunity, fatigue, and cognitive deficits (8–10). Also, altered serum Se levels have been documented in both autism and schizophrenia (11, 12), and it is hypothesized that redox imbalance during neurodevelopment increases risk for these neuropsychiatric conditions (13, 14).

On the opposite end of the spectrum, Se-excess can lead to toxicity and increased oxidative stress. In rodents, acute Se overexposure elicits motor deficits, catalepsy-like behavior and increased levels of dopamine, with inorganic selenium compounds being significantly more toxic than organic counterparts (15, 16). Moreover, in humans, rare cases of chronic Se-excess have been associated with elevated incidences of amyotrophic lateral sclerosis (17–19). Additionally, elevated selenium intake has been linked to higher incidences of type 2 diabetes (20), as have heightened levels of the selenium transport protein, selenoprotein P (21).

Whereas, the influence of Se has been extensively studied in many contexts, the developmental *in vivo* effects of chronic Se-deficiency and Se-excess upon measures of neurobehavior and energy metabolism have not been comprehensively characterized. Thus, we performed an expansive assessment of various behavioral and metabolic indices in young adult mice receiving dietary supplementation at levels corresponding to Se-deficient, Se-supplemented, and Se-excess upon weaning.

## Materials and Methods

### Animals

All experiments were conducted on male C57BL/6J mice purchased from Jackson labs at 3–4 weeks of age. Mice were maintained on a 12-h light/dark cycle and provided *ad libitum* food and water access. Procedures and experimental protocols were approved by the University of Hawaii's Institutional Animal Care and Use Committee. All efforts were made to minimize animal discomfort and number of animals used.

### Diet

Upon arrival at the University of Hawaii Animal Facility, mice were allocated into three groups, representing conditions of Se-deficiency, Se-supplementation, and Se-excess. All mice were administered Se-deficient laboratory chow (~0.08 ppm Se) (Research Diets, D19101Y), for which casein is the main source of both protein and Se, and the predominant Se species are organic. The Se-supplemented and Se-excess groups received sodium selenite in the drinking water at doses of 10 μM and 100 μM, respectively. Hundred μM sodium selenite corresponds to ~ 8 ppm elemental Se, a dosage reported to induce mortality in rats (22) and elicit clinical symptoms in humans (23).

### Experimental Design

Mice were group-housed until 10 weeks of age and then single-housed 3 days prior to onset of behavioral experiments. Spatial learning was assessed on the Barnes maze at 10–12 weeks of age, followed by metabolic phenotyping at 14–16 weeks. Motor coordination was periodically examined at 8, 12, and 16 weeks of age. Testing for acoustic startle/prepulse inhibition was performed last, at 17–18 weeks, as this procedure involves loud auditory stimuli and could potentially confound other behavioral procedures. At 20 weeks of age, mice were euthanized via CO_2_ asphyxiation for collection of fresh tissue or deeply anesthetized (1.2% Avertin; 0.7 ml/mouse) and perfused with 4% paraformaldehyde for immunohistology. Blood was also collected upon sacrifice, and in non-perfused mice, fat depots for gonadal and inguinal white adipose tissue were collected and weighed. Brains from non-perfused mice were split along the sagittal plane, with one hemisphere used for Se analysis and the other hemisphere allocated for biochemical assays.

### Barnes Maze Test

Spatial learning was assessed using the Barnes maze (TSE Systems) as described previously (24). In brief, the maze consists of a white circular board containing 40 equally spaced holes, with one hole leading to an escape tunnel. Mice were trained to find the escape tunnel, which remained at a fixed location relative to spatial cues for the duration of training. Training consisted of two trials daily (3 min max per trial) for 10 days, with the starting location varying pseudorandomly among the four quadrants. If a mouse failed to find the escape tunnel within the 3 min trial period, it was placed in the escape tunnel by the researcher and allowed to stay there for 15 s. For each training trial, the latency to locate the escape tunnel and the number of incorrect holes checked (errors) before locating the escape tunnel were recorded. For analysis purposes, data were grouped into trial blocks, which consisted of 4 trials, with each trial administered from a distinct quadrant.

### Rotarod Test

Starting speed for the Rotarod was 4 rpm and increased to 40 rpm over a 5 min period. The latency to fall off the rod was measured for each trial and the best score for each mouse was used for statistical analysis.

### Acoustic Startle and Prepulse Inhibition

Mice were placed in the startle chamber (Responder-X, Columbus Instruments, Columbus, OH) and allowed a 5-min acclimation period with the background noise (70 dB) continually present. Following acclimation, two blocks of trials were administered to assess the acoustic startle response and prepulse inhibition, respectively, as described previously (25).

### Glycemic Control Testing

Glucose tolerance was assessed by administering a glucose injection of 1 mg/g of body weight to animals that were fasted overnight. Tail blood was collected at time points 0, 30, 60, 120, and 180 min after injection and glucose levels were determined using strips and a glucometer (OneTouch Ultra, Lifescan).

### Lipid Droplet Analysis of Brown Adipose Tissue (BAT)

BAT was collected from perfused animals (*n* = 4 per group), embedded in paraffin, sectioned at 5 μm, and stained with hematoxylin and eosin. Bright field images were taken at 20× magnification and imported into FIJI for image analysis. Images were thresholded, and droplets were measured using the “Analyze Particles” feature of FIJI. For each subject, 500–700 lipid droplets were measured.

### Metabolic Chambers

Locomotion, respiratory metabolism, and ingestive behavior were measured using the PanLab Oxylet*Pro*^TM^ System (Harvard Apparatus, Barcelona, Spain) according to the manufacturer's instructions. Mice were placed in individual chambers, with fresh bedding, food, and water, and allowed to acclimate for 24 h, followed by 48 h of data collection. Cage air was sampled for 7 min periods every 35 min to measure oxygen and carbon dioxide concentrations. Data were collected and analyzed with Panlab METABOLISM software (Vídenská, Prague, Czech Republic).

### Protein Extraction and Immunoblotting

Frozen tissues were lysed by sonication in CelLytic MT buffer (Sigma-Aldrich) containing protease inhibitors (Calbiochem) and centrifuged at 14,000 *g* for 10 min at 4°C. Supernatants were collected and the protein concentrations were measured using the Bradford assay. For western blotting, 40 μg samples of total protein were separated on 4–20% SDS-PAGE gradient gels (Bio-Rad), transferred to Immobilon-FL polyvinylidene difluoride membranes (Millipore), and probed for 2 h at room temperature with specific antibodies. Membranes were then incubated in the dark with secondary antibodies coupled to infrared fluorophores (LI-COR Biosciences). Blots were imaged and analyzed using an Odyssey infrared imager (LI-COR Biosciences). Relative protein levels were determined by dividing the optical density of the band representing the protein of interest by that of the loading control (β-actin).

### Antibodies

Primary antibodies used for Western blotting were as follows: goat anti-GPX1 (1:500; R&D Systems, AF3798), mouse anti-SELENBP1 (1:1,000, MBL, M061-3), rabbit anti-TXNRD2 (1:1,000; Invitrogen, LF-PA0024), and rabbit anti-β-actin (1:5,000; Cell Signaling, 4970S).

### Leptin ELISA

Serum leptin levels were measured using a commercially available solid-phase sandwich ELISA kit (Invitrogen) according to the manufacturer's instructions.

### Se Analysis

Se was measured using a modification of the fluorometric assay of Koh and Benson (26) and Sheehan and Gao (27). Tissue was predigested in 6 ml nitric acid at 150–300°C for 2 h. Hundred μl predigested tissue, serum, or Se standard (Millipore Sigma, 89598) was then digested with 0.5 ml perchloric:nitric acid (1:4) at 197° for 1.5 h. As samples cooled to 150°C, 0.5 ml hydrochloric acid (HCl) was added and samples were maintained at 130–150°C for 30 min. Next, 2 ml 0.1 M EDTA, 0.5 ml 2,3 diaminonaphthalene (0.1% w/v in 0.1 M HCl), and 3 ml cyclohexane were added, followed by incubation at 60°C for 30 min. Fluorescence was measured in a Perkin-Elmer LS 55 fluorometer and concentrations determined via comparison to a standard curve.

### Glutathione Peroxidase Activity Assay

Soluble proteins were extracted as described above and normalized to a concentration of 4 mg/ml. Glutathione peroxidase activity was measured as the reduction rate of cumene hydroperoxide catalyzed by the samples upon oxidation of nicotinamide adenine dinucleotide phosphate (NADPH) using a commercially available kit (Cayman Chemical). A unit of activity was defined as the consumption of 1 μmol of NADPH per min, calculated from the expression (*V*_*max*_ X *V*_*t*/_*V*_*s*_)/(0.0062 X D), using 0.0062 μM^−1^ cm^−1^ as the extinction coefficient for NADPH at 340 nm.

### Statistical Analysis

Data were analyzed and plotted using Prism software (GraphPad). Statistical tests varied according to the experiment and are indicated in the text and/or figure legends. To compare multiple groups, ANOVAs and Tukey's *post-hoc* test were utilized. All results are represented as mean ± standard error of the mean (SEM).

## Results

**Figure 1:**
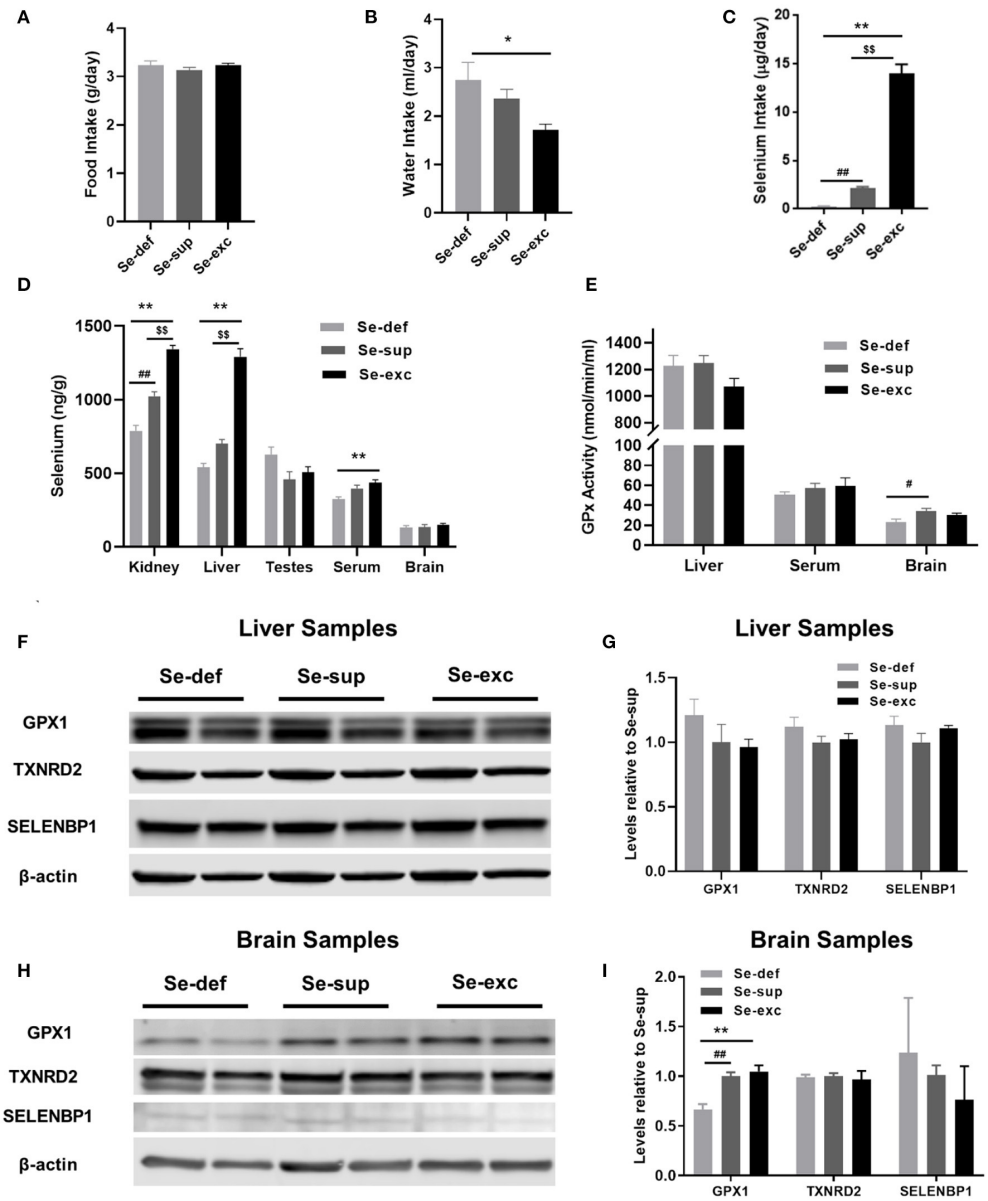
Assessment of varying Se supplementation on organ-specific Se content, glutathione peroxidase activity, and selenoprotein levels. **(A)** Mean (±SEM) daily food intake. **(B)** Mean (±SEM) daily water consumption. **(C)** Mean (±SEM) daily selenium intake (*n* = 6–7 per group). **(D)** Mean (±SEM) selenium content in kidney, liver, testes, serum, and brain (*n* = 3–4 per group). **(E)** Mean (±SEM) GPX activity in liver, serum, and brain (*n* = 4 – 6). **(F–I)** Protein levels of GPX1, TXNRD2, and SELENBP1 in liver **(F,G)** and brain **(H,I)** (*n* = 4). ^$$^*p* < 0.01 between Se-exc and Se-sup groups; ^*^p < 0.05 between Se-exc and Se-def groups; ^**^p < 0.01 between Se-exc and Se-def groups; ^#^*p* < 0.05 between Se-def and Se-sup groups; ^##^*p* < 0.01 between Se-def and Se-sup groups.

For this study, newly weaned male mice were allocated into three groups devised to represent conditions of Se-deficiency (Se-def), Se-supplementation (Se-sup), and Se-excess (Se-exc). All mice were fed Se-deficient laboratory chow (~0.08 ppm Se), with the Se-sup and Se-exc groups receiving additional Se supplementation in their drinking water at doses of 10 μM and 100 μM, respectively. As anticipated, we observed no differences among groups for food intake (Figure 1A), although water consumption did vary (Figure 1B) [*F*_(2, 17)_ = 4.918, *p* = 0.0206], with significant differences between the Se-def and Se-exc groups (*p* = 0.018). We also calculated Se intake based on water and food consumption, and mean values corresponded to 0.26, 2.14, and 13.94 μg/days for the Se-def, Se-sup, and Se-exc groups, respectively (Figure 1C).

Upon sacrifice, tissue was harvested for determination of Se content and additional molecular analyses. For kidney [*F*_(2, 9)_ = 78.91, *p* < 0.0001], liver [*F*_(2, 9)_ = 100.5, *p* < 0.0001], and serum samples [*F*_(2, 9)_ = 8.578, *p* = 0.0082], Se levels differed among groups in a dose-dependent manner, whereas in brain [*F*_(2, 8)_ = 0.7685, *p* = 0.4951] and testes [*F*_(2, 9)_ = 3.430, *p* = 0.0781], levels were comparable (Figure 1D). Parallel analyses of glutathione peroxidase (GPx) activity were conducted on liver, serum, and brain samples. Surprisingly, liver GPx activity [*F*_(2, 9)_ = 2.256, *p* = 0.1606] was similar between groups, whereas serum GPx activity [*F*_(2, 9)_ = 0.5429, *p* = 0.5937] showed similar non-significant trends as observed for Se analysis (Figure 1E). For brain, we detected a significant main effect of Se group upon GPx activity [*F*_(2, 9)_ = 4.263, *p* = 0.0498], with differences between the Se-def and Se-sup groups attaining significance (*p* = 0.0441). Western blotting was also performed on liver and brain samples to assess various markers of Se status. We probed for GPX1 and TXNRD2, two abundant selenoproteins known to be responsive and non-responsive to alterations in Se supply (28), respectively, and the selenium binding protein (SELENBP1), a putative factor protecting against Se-toxicity (29). For liver samples, levels of GPX1, TXNRD2, and SELENBP1 were not impacted by Se group (Figures 1F,G). Brain levels of TXNRD2 and SELENBP1 were comparable between groups, but we did observe altered levels of GPX1 [*F*_(2, 9)_ = 15.67, *p* = 0.0012], as levels were significantly reduced in the Se-def group (vs Se-sup: *p* = 0.0038; vs. Se-exc: *p* = 0.0016) (Figures 1H,I).

**Figure 2:**
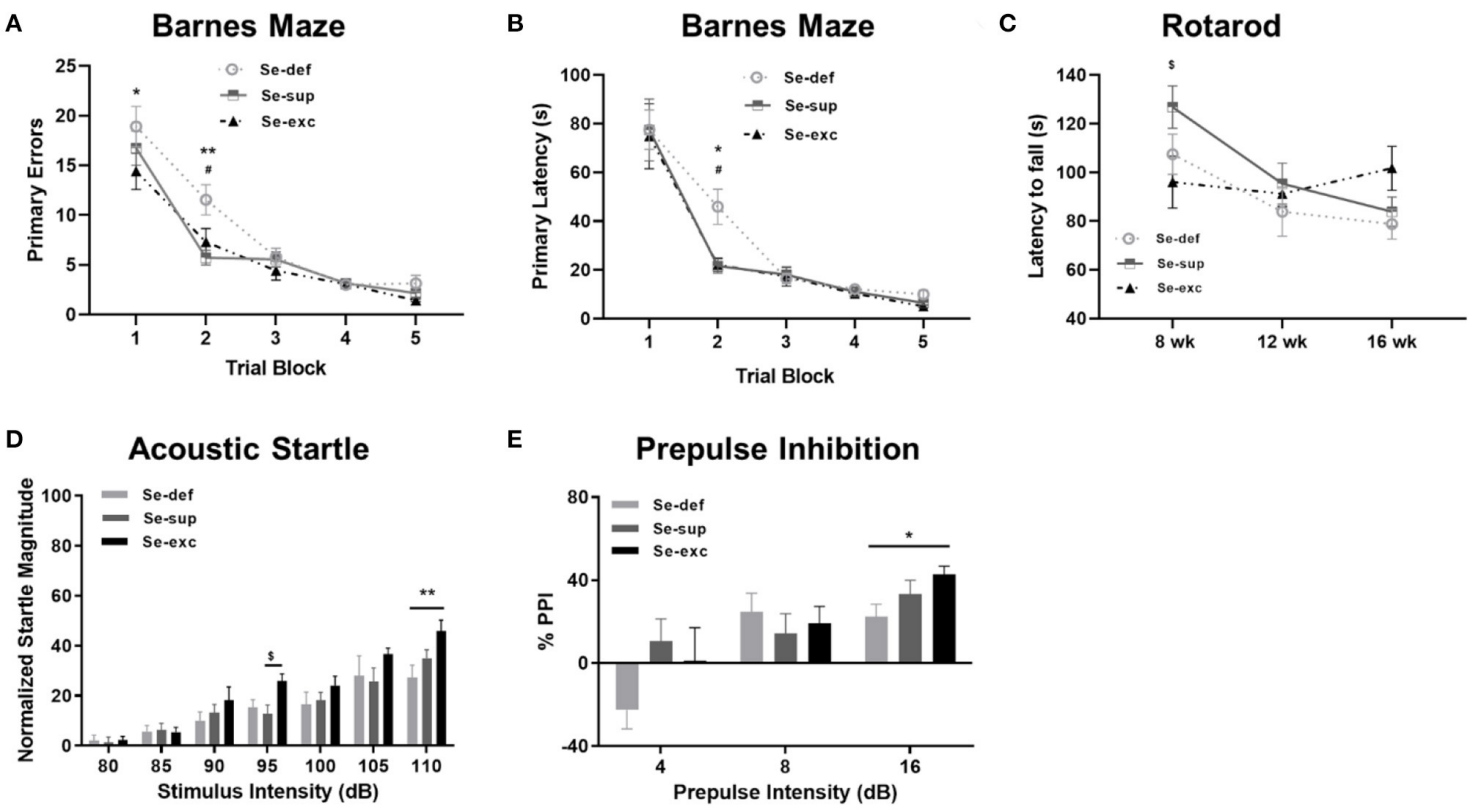
Se-deficient mice exhibit deficits in cognition and sensorimotor gating. **(A)** Mean (±SEM) number of incorrect holes checked before locating the escape tunnel during Barnes maze training. **(B)** Mean (±SEM) latency to locate the escape tunnel during maze training. **(C)** Mean (±SEM) latency to fall off the Rotarod at 8, 12, and 16 weeks of age. **(D)** Mean (±SEM) normalized startle magnitude as a function of acoustic startle intensity. **(E)** Mean (±SEM) percentage of prepulse inhibition as a function of prepulse intensity above the background level (70 dB). ^$^*p* < 0.05 between Se-exc and Se-sup groups; *p < 0.05 between Se-exc and Se-def groups; **p < 0.01 between Se-exc and Se-def groups; ^#^*p* < 0.05 between Se-def and Se-sup groups (*n* = 9–10 animals per group for all experiments).

Prior to harvesting of tissue, mice were subjected to a battery of neurobehavioral and metabolic tests. Cognition was evaluated using the Barnes maze, a widely utilized paradigm for spatial learning in rodents. Mice were trained to find a hidden escape tunnel located beneath one of 40 holes on the periphery of the circular maze. As anticipated, we observed a main effect of time on spatial learning, as indicated by less primary errors [*F*_(5, 100)_ = 88.6, *p* < 0.001] and a faster primary latency (*F*_(5, 100)_ = 66.7, *p* < 0.0001] when locating the escape tunnel (Figures 2A,B). We also detected a main effect of Se group upon the number of primary errors [*F*_(2, 25)_ = 4.907, *p* = 0.0159], but not upon the primary latency [*F*_(2, 25)_ = 1.44, *p* = 0.2560]. For both primary errors [*F*_(8, 100)_ = 1.697, *p* = 0.1083] and primary latency [*F*_(8, 100)_ = 1.015, *p* = 0.4295], the time × Se group interaction was non-significant. *Post-hoc* analyses revealed higher levels of primary errors in the Se-def group during trial block one (vs. Se-exc: *p* = 0.0151) and two (vs Se-sup: *p* =0.0008; vs. Se-exc: *p* = 0.0233). Likewise, primary latencies were significantly higher in the Se-def group during trial block 2 (vs Se-sup: *p* = 0.0146; vs. Se-exc: *p* = 0.0144). No significant differences were observed between groups for these measures during the remaining trial blocks nor during a probe trial conducted after trial block 5 (data not shown). As a whole, these results indicate that spatial learning is impaired by Se-deficiency.

Motor coordination, as determined by the latency to fall off a rotating rod of increasing speed, was examined at 8, 12, and 16 weeks of age (Figure 2C). Two-way ANOVA analyses revealed a main effect of time [*F*_(2, 50)_ = 7.826, *p* = 0.0011) and a significant time x Se group interaction effect [*F*_(4, 50)_ = 2.779, *p* = 0.0367], whereas the influence of Se group was non-significant [*F*_(2, 25)_ = 1.208, *p* = 0.3156]. In our initial test at 8 weeks of age, we observed differences between the Se-sup and Se-exc groups, with Se-exc mice performing significantly worse (*p* = 0.0252). Surprisingly, motor coordination improved over time in the Se-exc group, whereas performance declined in both the Se-def and Se-sup groups.

To assess sensorimotor gating, mice were tested for acoustic startle reactivity and prepulse inhibition. For acoustic startle (Figure 2D), we observed a main effect for stimulus intensity [*F*_(6, 150)_ = 53.31, *p* < 0.0001), whereas both Se group [*F*_(2, 25)_ = 2.241, *p* = 0.1273] and the stimulus intensity × Se group interaction [*F*_(12, 150)_ = 1.610, *p* = 0.0942] were not significant. Across the vast majority of stimulus intensities, startle magnitude was most pronounced in the Se-exc group, with statistically significant differences detected at 95 dB (vs. Se-sup: *p* = 0.0410) and 110 dB (vs. Se-def: *p* = 0.0025). In testing for prepulse inhibition (Figure 2E), a main effect of prepulse intensity [*F*_(2, 50)_ = 16.07, *p* < 0.0001] was found, while the effects of Se group [*F*_(2, 25)_ = 1.062, *p* = 0.3608] and the prepulse intensity × Se group interaction [*F*_(4, 50)_ = 2.185, *p* = 0.0841] failed to reach significance. Moreover, at the highest prepulse intensity (16 dB), the Se-def group exhibited significantly reduced inhibition relative to the Se-exc group (*p* = 0.0361).

**Figure 3:**
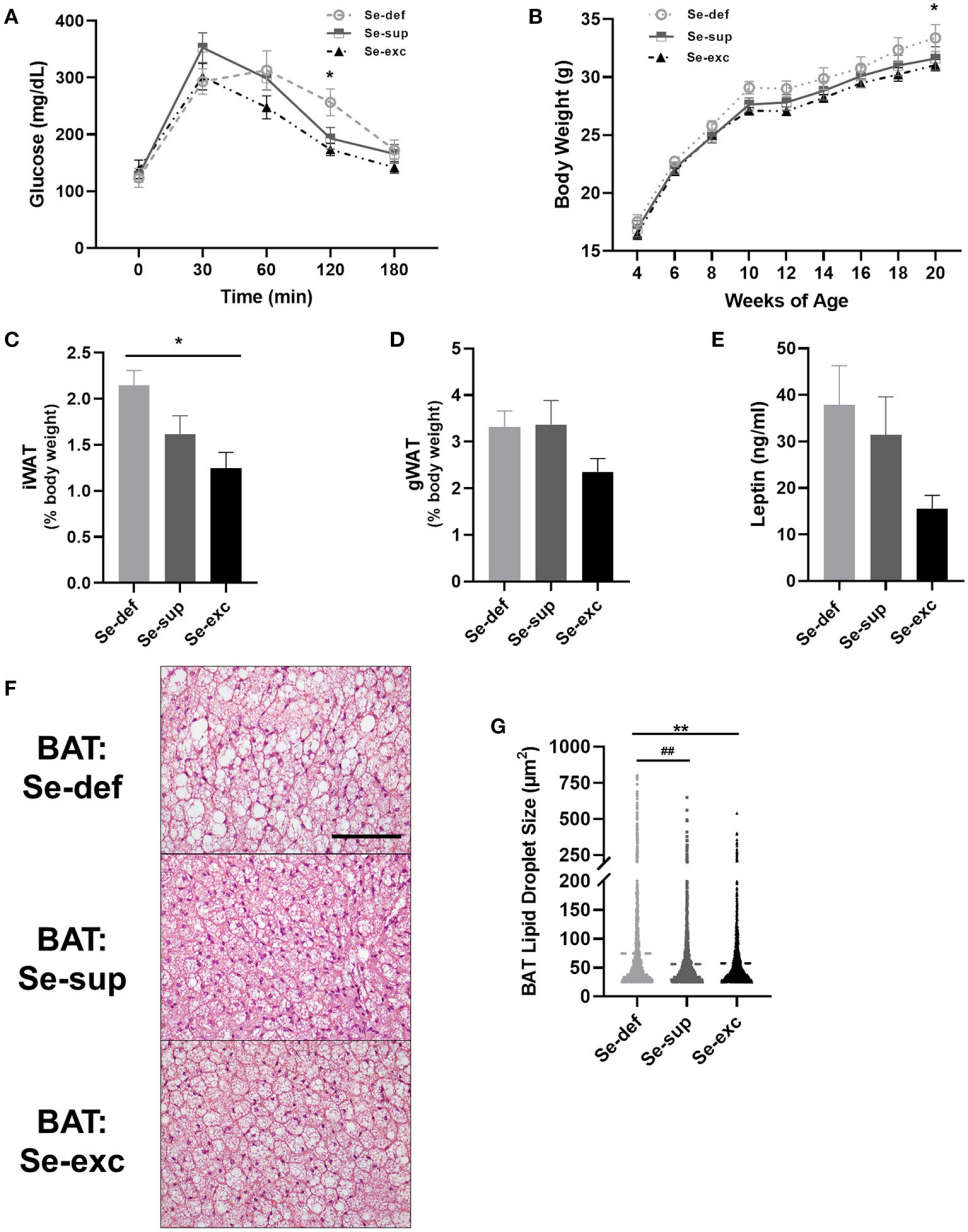
Impaired glucose tolerance and increased adiposity in Se-deficient mice. **(A)** Mean (±SEM) blood glucose levels during glucose tolerance testing (*n* = 7–8 per group). **(B)** Mean (±SEM) body weight from 4 to 20 weeks of age (*n* = 9–10). **(C)** Mean (±SEM) inguinal white adipose tissue (*n* = 4). **(D)** Mean (±SEM) gonadal white adipose tissue relative to total body weight (*n* = 4). **(E)** Mean (±SEM) serum leptin levels (*n* = 7–9). **(F)** Representative images of brown adipose tissue (BAT). **(G)** Scatter plot of BAT lipid droplet size (*n* = 4). Scale bar = 100 μm, *p < 0.05 between Se-exc and Se-def groups, **p < 0.01 between Se-exc and Se-def groups; ^##^*p* < 0.01 between Se-def and Se-sup groups.

Mice were also tested for glycemic control and body weight was regularly monitored. For glucose tolerance testing (Figure 3A), a main effect of time [*F*_(4, 76)_ = 63.18, *p* < 0.0001] and a significant time x Se group interaction effect [*F*_(8, 76)_ = 2.454, *p* = 0.0203] was detected, whereas the effect of Se group was non-significant [*F*_(2, 19)_ = 1.680, *p* = 0.2129]. *Post-hoc* tests revealed significantly elevated blood glucose levels 120 min after glucose injection in the Se-def group relative to the Se-exc group (*p* = 0.0148). With respect to body weight, two-way ANOVA analysis revealed a main effect of time [*F*_(8, 200)_ = 316.1, *p* < 0.0001], with non-significant effects observed for Se group [*F*_(2, 25)_ = 2.307, *p* = 0.1203] and the time × Se group interaction [*F*_(16, 200)_ = 0.4277, *p* = 0.9740]. Levels gradually diverged over time between the Se-def and Se-exc groups, with differences reaching significance at 20 wks (Figure 3B) (*p* = 0.0443). Upon sacrifice, we also found that relative levels of inguinal white adipose tissue (iWAT) differed between groups (Figure 3C) [*F*_(2, 9)_ = 6.227, *p* = 0.0201], with statistically significant differences between the Se-def and Se-exc groups (*p* = 0.0165). Similar non-significant trends were also observed for gonadal white adipose tissue (gWAT) (Figure 3D) [*F*_(2, 9)_ = 2.119, *p* = 0.1762] and serum leptin (Figure 3E) [*F*_(2, 21)_ = 2.563, *p* = 0.1009]. Finally, average lipid droplet size in brown adipose tissue (BAT) significantly differed between groups (Figures 3F,G) [*F*_(2, 7, 750)_ = 77.77, *p* < 0.0001], as droplets were larger in the Se-def group (*p* < 0.0001).

**Figure 4:**
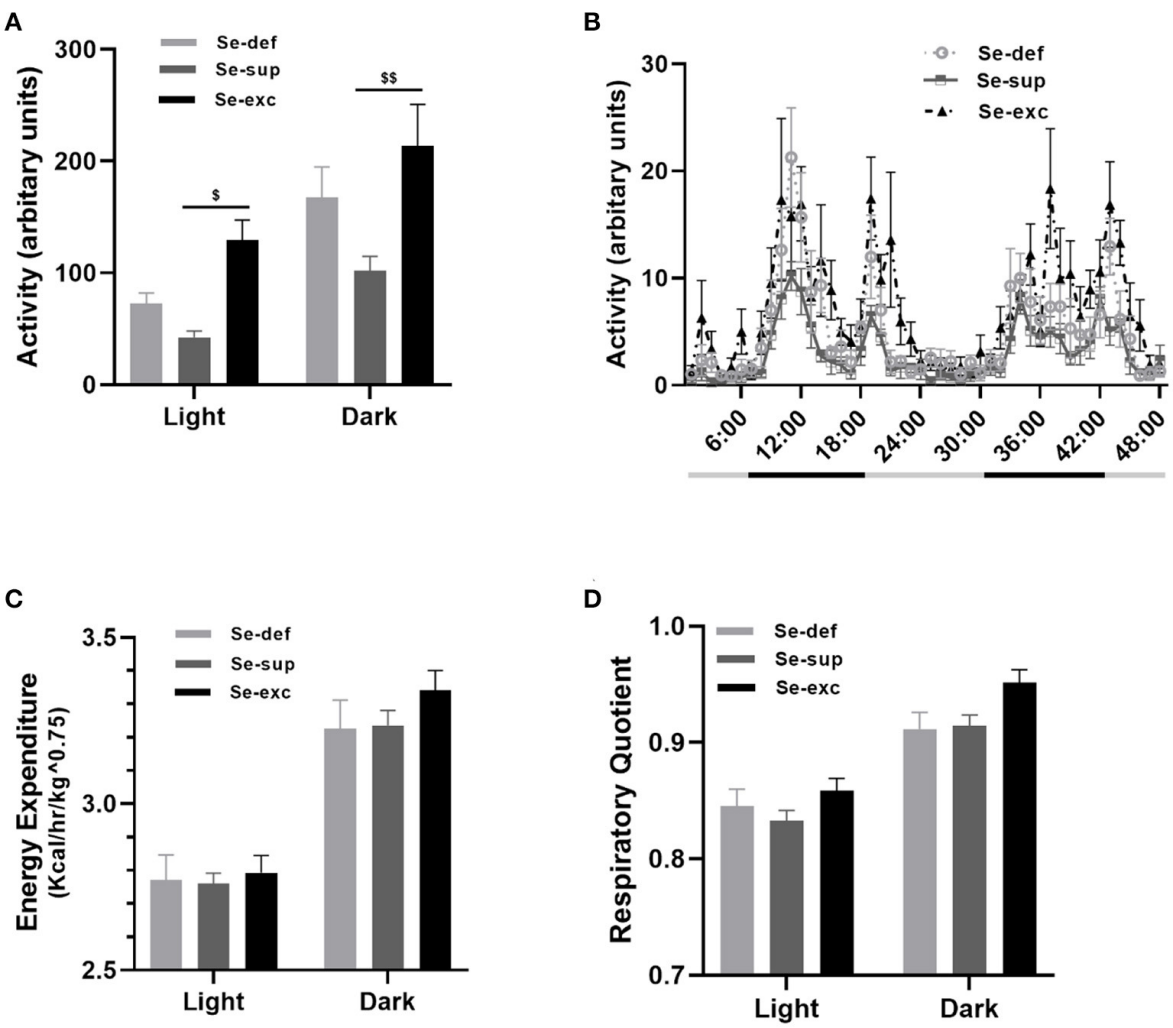
Influence of varying Se supplementation on locomotion and respiratory metabolism. **(A)** Mean (±SEM) locomotor activity during the light and dark cycles. **(B)** 48-h time course of locomotor activity. **(C)** Mean (±SEM) energy expenditure during the light and dark cycles. **(D)** Mean (±SEM) respiratory quotient during the light and dark cycles. ^$^*p* < 0.05 between Se-exc and Se-sup groups; ^$$^*p* < 0.01 between Se-exc and Se-sup groups; (*n* = 6–7 animals per group for all experiments).

At 14–16 weeks of age, mice were placed in metabolic chambers for 48-hrs to evaluate activity, respiratory metabolism, and ingestive behavior. For locomotion (Figures 4A,B), two-way ANOVA analysis detected main effects for both light cycle [*F*_(1, 17)_ = 46.81, *p* < 0.0001] and Se-group [*F*_(2, 17)_ = 7.126, *p* = 0.0057], in conjunction with a non-significant light cycle x Se group interaction effect [*F*_(2, 17)_ = 0.809, *p* = 0.4617]. During both the light (*p* = 0.0164) and dark cycles (*p* = 0.0018), the Se-exc group exhibited elevated locomotion relative to the Se-sup group. For measures of energy expenditure (Figure 4C) [EE: *F*_(1, 17)_ = 1,238, *p* < 0.0001] and the respiratory quotient (Figure 4D) [RQ: *F*_(1, 17)_ = 140.2, *p* < 0.0001], we also observed a main effect for the light cycle, but not for Se group [EE: *F*_(2, 17)_ = 0.5002, *p* = 0.6151; RQ: *F*_(2, 17)_ = 3.126, *p* = 0.0698]. Moreover, we also detected a significant light cycle x Se group interaction effect for energy expenditure [EE: *F*_(2, 17)_ = 4.133, *p* = 0.0344], but not for the respiratory quotient [RQ: *F*_(2, 17)_ = 1.212, *p* = 0.3222].

## Discussion

In summary, these results detail the negative consequences of juvenile Se-deficiency upon measures of behavior and metabolism in early adulthood. Se-deficient mice displayed delayed learning and altered sensorimotor gating, and these deficits coincided with reduced GPx activity in brain. Moreover, Se-deficiency also resulted in impaired glycemic control, elevated body weight, and increased adiposity. Finally, Se-excess, at levels known to be toxic to humans, was surprisingly well-tolerated in mice and exerted beneficial effects on energy metabolism.

Our study corroborates prior findings that Se-deficiency hinders spatial learning (30, 31) and, to the best of our knowledge, represents the first association of Se-deficiency with impairments in sensorimotor gating. Deficits in cognition and sensorimotor gating are hallmarks of many neurodevelopmental disorders, including schizophrenia and autism. For both schizophrenia (11, 32, 33) and autism (12, 34, 35), reduced Se levels have been chronicled in the literature, albeit there are many exceptions (36–38), and it is unclear whether this represents a cause or consequence of these conditions. Of particular significance to our results is a recent report examining the Se status of 287 Polish children, which were divided into four groups, corresponding to: (1) autism spectrum disorder (ASD) with obesity, (2) ASD without obesity, (3) non-ASD with obesity, and (4) non-ASD without obesity (12). Observed Se levels were lowest in ASD patients with obesity and highest in non-ASD patients without obesity, with differences between groups being highly significant (*p* < 0.001) for serum, urine, toenail samples. Moreover, across groups, Se levels were inversely correlated with body mass index (p < 0.001) for all sample types.

The influence of Se supplementation upon energy metabolism is hotly debated and nuanced in the existing literature. An unanticipated corollary of the Nutritional Prevention of Cancer (NPC) trial was the observation that Se-supplementation (200 μg daily as Se-yeast) increased risk of type 2 diabetes for participants with baseline plasma Se levels within the upper tertile (20). Since these findings were documented, excess Se supplementation has been shown to adversely impact insulin signaling in multiple rodent models (39, 40). In contrast, reduced serum Se levels have been observed in morbidly obese patients (41), and supranutritional Se supplementation (240 μg/day) in the form of selenomethionine (SeMet) was recently shown to decrease both fat mass and circulating leptin levels in a 3-months dietary intervention study of obese individuals (42). Of potential relevance, we previously reported increased adiposity and elevated leptin levels in mice lacking SELENOM (43), an ER-resident selenoprotein that is highly expressed in brain and regulated by Se levels. Further studies showed that leptin upregulates SELENOM in hypothalamic neurons and that SELENOM, in turn, promotes leptin signaling (44). More recently, supranutrional Se supplementation (2.25 ppm SeMet in chow) was found to facilitate selenocysteine incorporation at sites canonically encoding cysteine, promote thermogenesis, and protect against diet-induced obesity (45).

One unexpected outcome of this study was the beneficial influence of Se at a dosage (8 ppm in water) originally hypothesized to elicit toxic effects. We chose to use chow that was mildly Se-deficient and provide further Se supplementation in the drinking water as selenite to the Se-sup and Se-exc groups. Inorganic Se species (selenite, selenate) are less readily absorbed by the intestine than organic counterparts (SeMet) (46–48), and are also significantly more toxic. For instance, the toxicity of selenite was found to be 53-fold greater than that of SeMet when administered intracerebroventricularly to rats (15). With specific regard to supplementation of inorganic Se species in drinking water, increased mortality was previously reported at levels >6 ppm, although lower Se dosages (2–3 ppm) did lead to decreased body weights (22). Similarly, chow containing Se at >5 ppm, has been shown to adversely impact growth and mortality in rodents (49) and pigs (50), with effects being more severe when selenite was the predominant Se species. Although relatively rare in humans, Se intoxication leads to loss of hair and nails, skin lesions, and nervous system abnormalities. A case study of Se toxicity in the Enshi district of China reported neurological defects in 18 of 22 subjects displaying signs of selenosis, and symptoms included hyperreflexia, convulsions, motor weakness, and hemiplegia (51). Moreover, blood Se levels in affected patients were observed to be roughly 100 times greater than subjects receiving a Se-adequate diet. Chronic Se overexposure has also been associated with an elevated risk of neurodegenerative disease, specifically amyotrophic lateral sclerosis (ALS). This linkage was first noted in 1977, when a cluster of ALS cases was reported in a seleniferous area of South Dakota (19) and further substantiated by increased incidences of ALS in an Italian population chronically exposed (1974–1988) to drinking water containing high levels of selenate (18). Of further significance, elevated levels of selenite have been reported in the cerebrospinal fluid (CSF) of newly diagnosed ALS patients (52).

Another unanticipated finding was that Se supplementation modulated GPx activity to a greater extent in brain than liver. Brain Se levels are typically lower than other organs and blood (53), with Se homeostasis in the nervous system being tightly regulated by the blood-brain barrier (BBB) (54). Se transport to brain is regulated by endothelial cell-mediated uptake of SELENOP via the lipoprotein-related receptor, ApoER2, at the BBB (54, 55). SELENOP is also expressed in astrocytes (56–58), especially those lining the BBB, and astrocyte-derived SELENOP is speculated to supply ApoER2-expressing neurons with Se within the parenchyma (59). In cases of severe Se-deficiency, it is known that the brain and testes preferentially retain Se at the expense of other organs, and this phenomenon is dependent upon SELENOP and ApoER2 (55, 60–62). It should be duly noted that our Se-deficient chow contained 0.08 ppm Se, several-fold higher than that of many Se deprivation studies, but still well below the 0.15 ppm minimum recommended for rodent diets by the AIN (63). Given that liver and kidney represent the primary sites of Se metabolism and excretion (64), respectively, the fact that supplementation most impacted Se content in these tissues was expected. The effect of supplementation on serum Se was less robust, suggesting that most Se was converted to excretory metabolites in liver, with a small fraction being incorporated into SELENOP. Moreover, it appears that our Se-deficient diet did not affect liver GPx activity, in line with prior findings by Sunde and colleagues showing that hepatic levels of GPx activity plateau when dietary Se is 0.09 ppm or greater (65). Furthermore, prior evidence suggests that supplementation at levels similar to our study can significantly impact brain GPx activity. For example, Whanger and colleagues reported that increasing dietary Se content from 0.1 to 4 ppm raised GPX activity by 32 and 77% in cortex and cerebellum, respectively (66).

It is imperative to note this study has several caveats that merit consideration. First, to reduce cost and animal usage, only male mice were used. Sex-specific differences in the biological effects of Se are well-documented in the literature (67–69), with males typically being more adversely impacted by deviations in Se intake. Second, experiments were conducted on young adult mice (3–5 months) and the possibility that long-term Se overexposure elicits neurodegenerative effects at later time points cannot be ruled out. Finally, given that our dietary intervention began shortly after weaning, it is probable that Se supplementation triggered developmental epigenetic adaptations to cope with Se-excess. Interestingly, Se-excess mice performed significantly worse in the initial rotarod test, but their performance improved over time, while that of the other two groups (Se-def, Se-sup) declined. It is quite possible that providing adult mice with Se at our chosen excessive dose may elicit detrimental toxic effects not observed in juveniles.

Nevertheless, these results detail the adverse effects of mild Se-deficiency and suggest that juvenile Se status is critical for optimal neurodevelopment. These findings may have important implications for future prevention and treatment of neurodevelopmental disorders where redox imbalance is a key characteristic.

## Data Availability Statement

The raw data supporting the conclusions of this article will be made available by the authors, without undue reservation.

## Ethics Statement

The animal study was reviewed and approved by University of Hawaii's Institutional Animal Care and Use Committee.

## Author Contributions

VK and MP designed the experiments. VK, AS, DT, CC, JP, and MP performed research. CW contributed reagents/analytic tools. MP analyzed data and wrote the paper. All authors contributed to the article and approved the submitted version.

## Conflict of Interest

The authors declare that the research was conducted in the absence of any commercial or financial relationships that could be construed as a potential conflict of interest.
