# Supplementary material for: Juvenile Selenium Deficiency Impairs Cognition, Sensorimotor Gating, and Energy Homeostasis in Mice
Source: Front Nutr. 2021 May 7;8:667587. doi: 10.3389/fnut.2021.667587 (PMC8138326; doi:10.3389/fnut.2021.667587)

Full Blot for Figure 1F: Initial Probe

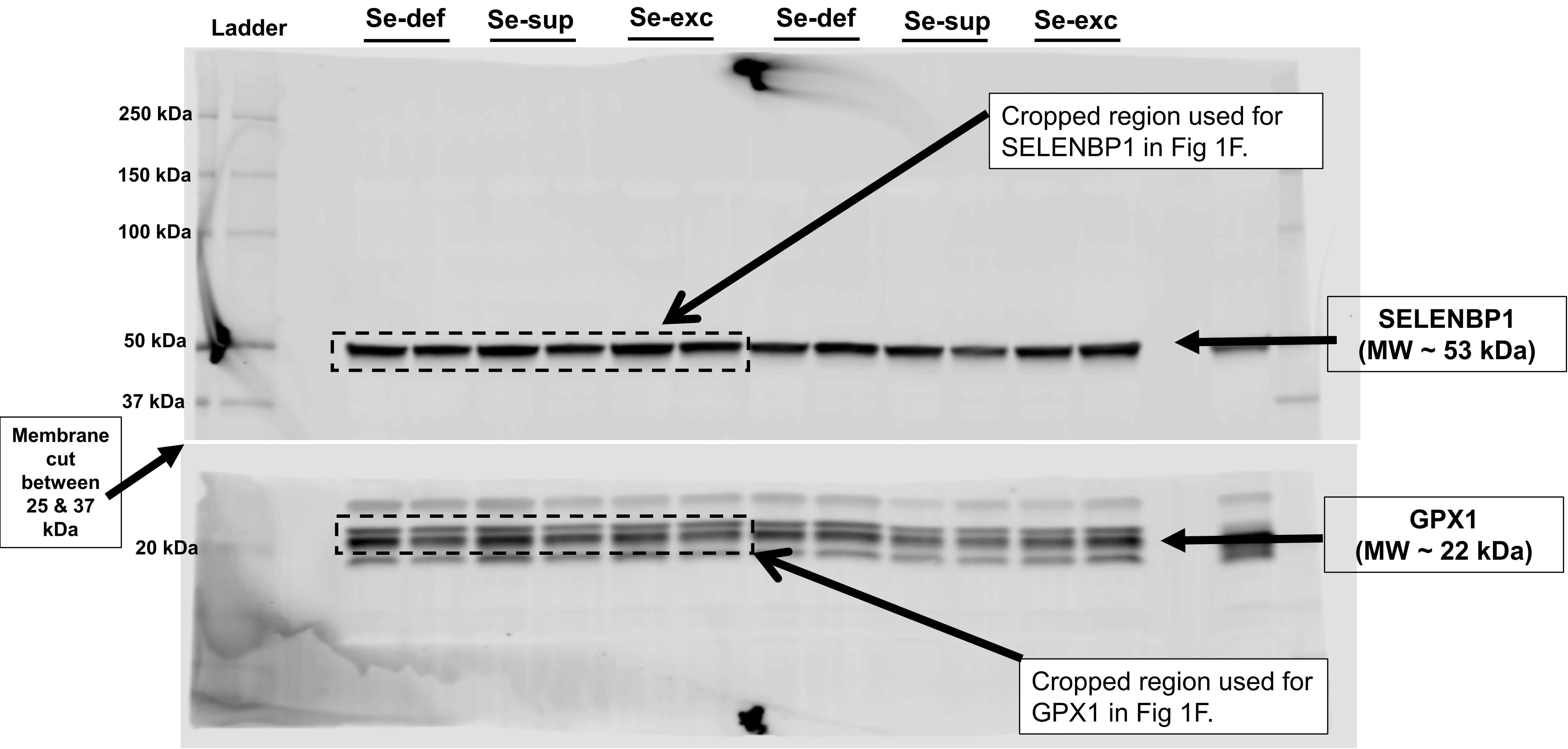

Full Blot for Figure 1F: Reprobe #1

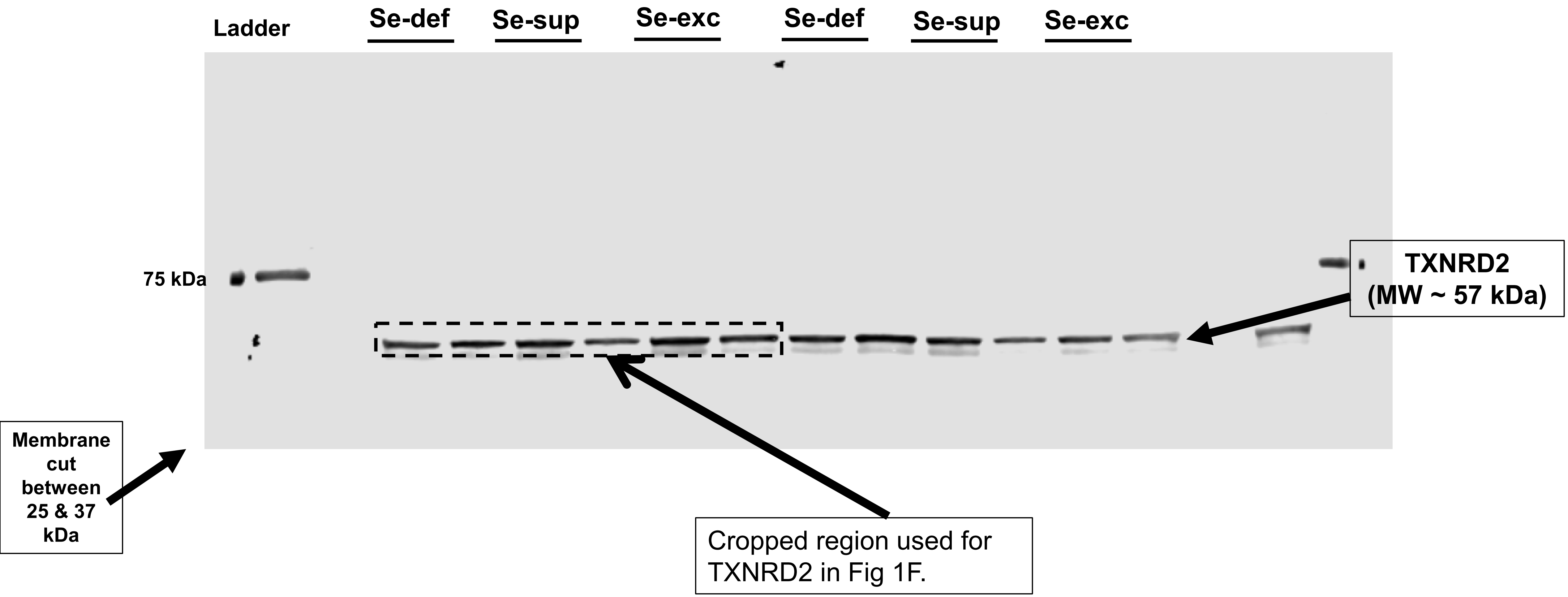

NOTE: TXNRD2 was detected in the 800 channel of the Licor Odyssey System. Only the 75 kDa band of the ladder was visible in this detection channel.

Full Blot for Figure 1F: Reprobe #2

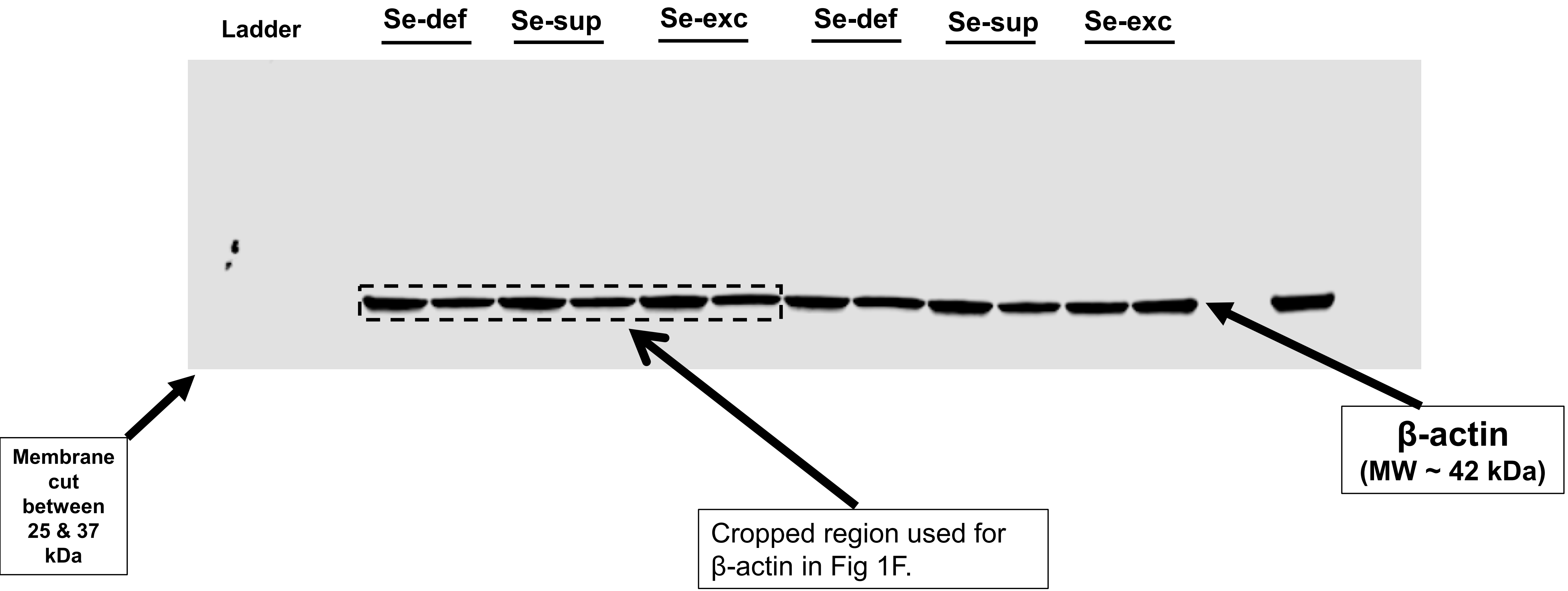

NOTE: β-actin was detected in the 800 channel of the Licor Odyssey System. Due to the intense signal of β-actin, the 75 kDa band of the ladder was not visible unless the signal was increased.

Full Blot for Figure 1H: Initial Probe (680 channel)

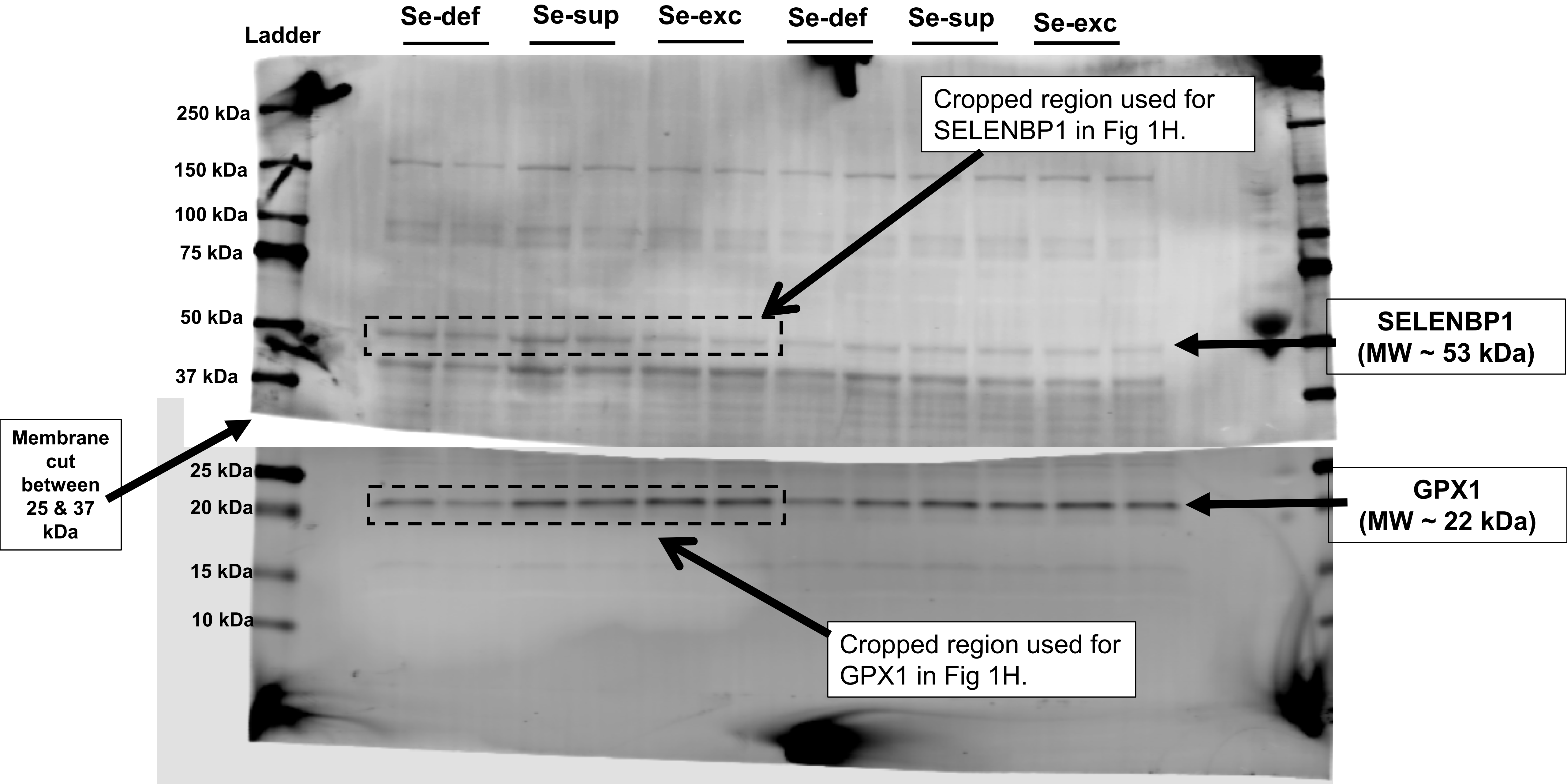

Full Blot for Figure 1H: Initial Probe (800 channel)

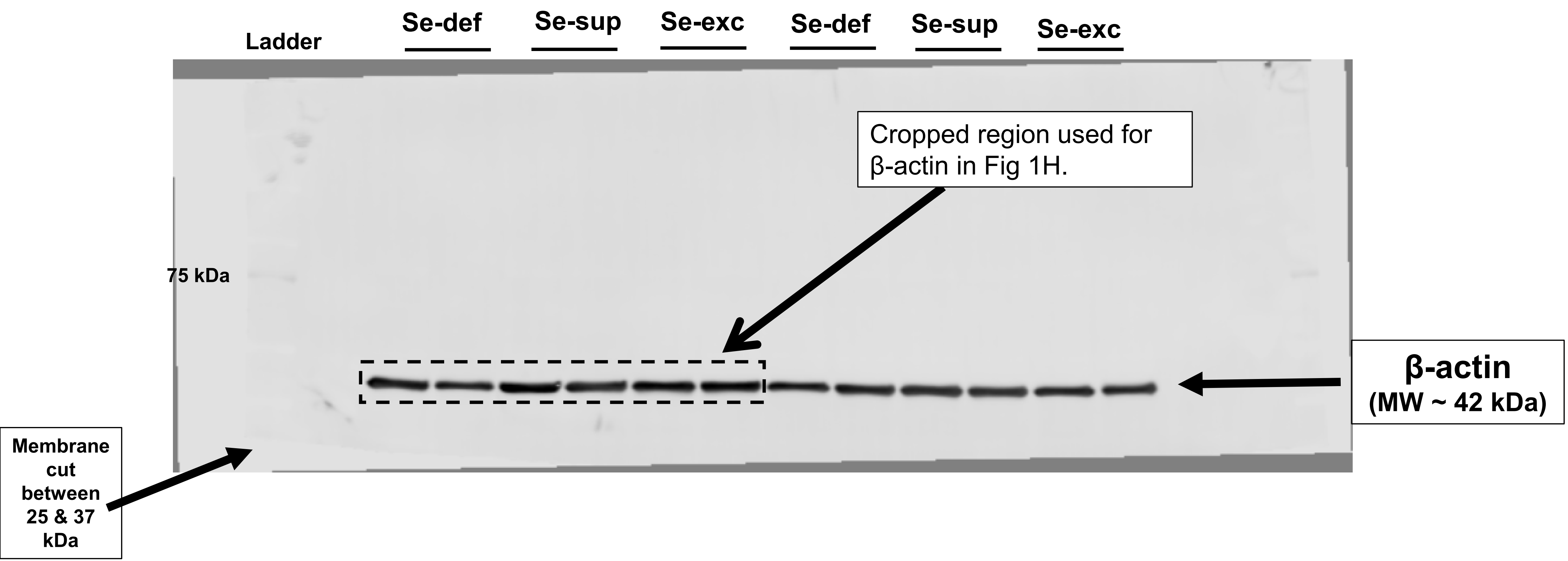

NOTE: β-actin was detected in the 800 channel of the Licor Odyssey System. Due to the intense signal of β-actin, the 75 kDa band of the ladder was barely visible.

Full Blot for Figure 1H: Reprobe #1

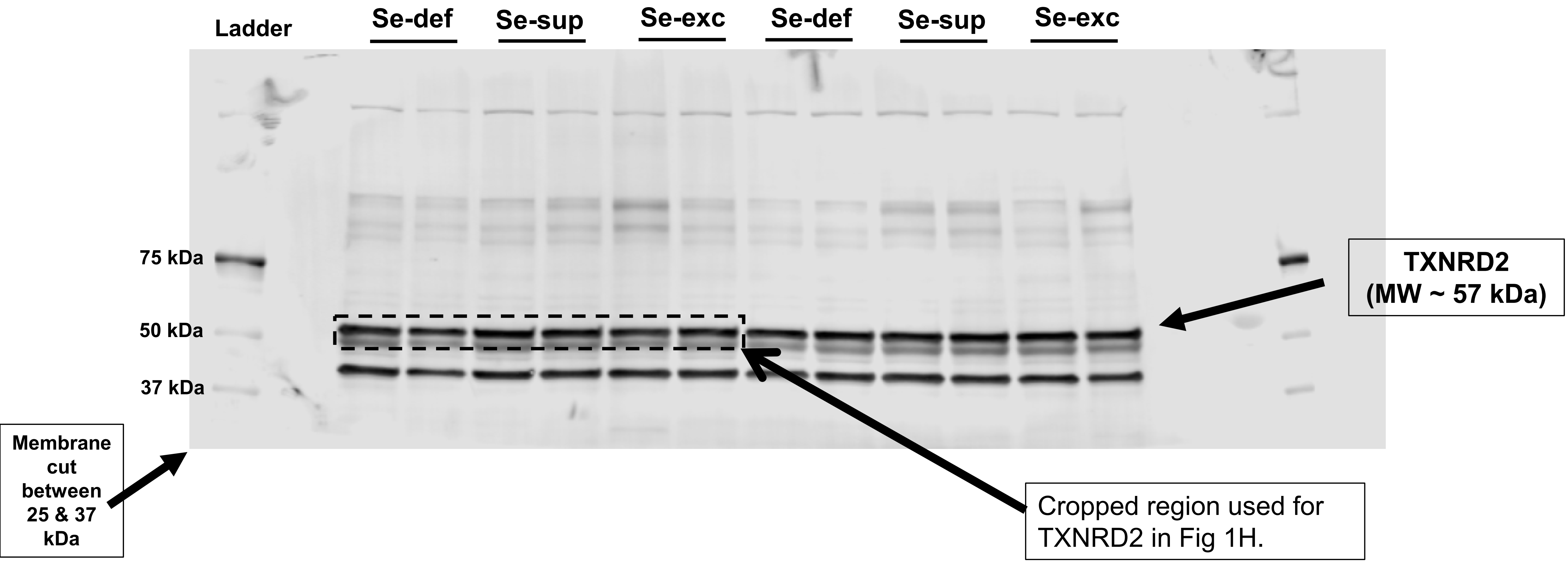

Supplement: Supplementary file 1 [file Image_1.pdf]
